# A small molecule induces integrin β4 nuclear translocation and apoptosis selectively in cancer cells with high expression of integrin β4

**DOI:** 10.18632/oncotarget.7646

**Published:** 2016-02-23

**Authors:** ShuYan Liu, Di Ge, LiNa Chen, Jing Zhao, Le Su, ShangLi Zhang, JunYing Miao, BaoXiang Zhao

**Affiliations:** ^1^ Shandong Provincial Key Laboratory of Animal Cells and Developmental Biology, School of Life Science, Shandong University, Jinan 250100, China; ^2^ School of Biological Science and Technology, University of Jinan, Jinan 250022, China; ^3^ Institute of Organic Chemistry, School of Chemistry and Chemical Engineering, Shandong University, Jinan 250100, China; ^4^ The Key Laboratory of Cardiovascular Remodeling and Function Research, Chinese Ministry of Education and Chinese Ministry of Health, Qilu Hospital, Shandong University, Jinan 250012, China

**Keywords:** chemical small molecules, integrin β4 high expression cancer, ANXA7, ATF3, apoptosis

## Abstract

Increased integrin β4 (ITGB4) level is accompanied by malignant progression of multiple carcinomas. However, selective therapeutic strategies against cancer cells expressing a high level of ITGB4 have not been reported. Here, for the first time, we report that a chiral small molecule, SEC, selectively promotes apoptosis in cancer cells expressing a high level of ITGB4 by inducing ITGB4 nuclear translocation. Nuclear ITGB4 can bind to the *ATF3* promoter region and activate the expression of *ATF3*, then upregulate the downstream pro-apoptosis genes. Furthermore, SEC promoted the binding of annexin A7 (ANXA7) to ITGB4 and increased ANXA7 GTPase activity. Activated ANXA7 promoted ITGB4 nuclear translocation by triggering ITGB4 phosphorylation at Y1494. SEC also inhibited the growth of xenograft tumors in the avian embryo model. We identified a small molecule, SEC, with selective pro-apoptosis effects on cancer cells with high expression of ITGB4, both *in vitro* and *in vivo*, by triggering the binding of ITGB4 and ANXA7, ITGB4 nuclear trafficking, and pro-apoptosis gene expression.

## INTRODUCTION

Integrin β4 (ITGB4) is the structural component of hemidesmosomes (HDs) that maintains epithelial architecture and also acts as a signaling adaptor driving tumor cell proliferation and movement [1, 2]. The two contrasting roles of ITGB4 in stable adhesion and pro-invasion are significantly correlated to its subcellular distribution. In normal epithelial cells, ITGB4 is incorporated in HDs, which contributes to the anchor of epothelial cells to the basal membrane. In carcinoma cells, ITGB4 redistributes from HDs to the leading edges of cells enriched at lamellipodia and filopodia and promotes tumor migration and invasion [3, 4]. Palmitoylation of ITGB4 cysteine residues results in ITGB4 mobilizing to lipid rafts. Lipid raft localization of ITGB4 is necessary for its binding to palmitoylated Src family kinase (SFK) and promoting EGF-dependent proliferation [5]. Hypoxia-induced recruitment of ITGB4 to rafts and the following internalization into multivesicles resulted in a shift from adhesion to metastasis [6].

This behavior of promoting tumor progression explains the upregulation of ITGB4 in multiple cancer cells and suggests that ITGB4 redistribution affords cells with advantages for proliferation and invasion. However, the growth-inhibitory effect of ITGB4 in carcinoma cells is not clear. The relevance of integrins in cancer progression has prompted the production of integrin β1 inhibitors, ATN-161 [7]. So far, no drugs are selective for ITGB4 in tumor therapy [8]. The identification of compounds that are highly selective for ITGB4 high-expressing cancer cells has been advancing but challenging.

We have been investigating the roles of ITGB4 in vascular endothelium cell (VEC) apoptosis. Increased ITGB4 level was accompanied by VEC apoptosis elicited by atorvastatin [9], deprivation of growth factors [10], safrole oxide [11], and a complex of copper and salicylaldehyde [12]. A derivative of isochroman (ISO-9) and low concentrations of cadmium inhibited VEC apoptosis by decreasing the level of ITGB4 [13, 14]. We found that ITGB4 level in VECs was very low in the presence of serum and fibroblast growth factor 2 (FGF-2) and was increased with their absence [10]. A small molecule, ECPC, promoted VEC apoptosis by stimulating ITGB4 nuclear translocation when ITGB4 was upregulated by deprivation of serum and FGF-2 [15]. However, the mechanisms by which ITGB4 enters the nucleus and triggers pro-apoptosis gene expression are not clear.

ITGB4 is distinguished by its large cytoplasm domain containing several phosphorylation sites at serine and tyrosine residues, which is essential for ITGB4 to be signaling-competent. Protein kinase C-α (PKCα) accounts for the phosphorylation of key serine residues (S1356, S1360, S1364), which contributes to HD disassembly [16, 17]. The phosphorylation of multiple ITGB4 tyrosine residues is mainly responsible for signaling transduction. Y1440 and Y1526 phosphorylation recruits Shc and thereby activates Ras-extracellular signal-regulated kinase (Ras-ERK) signaling [18]. ITGB4 Y1494 controls anchorage-independent tumor growth by activating ERK1/2 signaling and invasion by activating PI3K and Src [19]. Y1494 has been identified as a master regulator of ITGB4 phosphorylation and signaling. Mutation of Y1494 suppressed overall tyrosine phosphorylation of ITGB4 subunit [20].

Annexin A7 (ANXA7) is a Ca^2+^-dependent, phospholipid-binding protein and unique for its extraordinary long amino terminus [21]. ANXA7 is predominantly localized in cytoplasm, and translocates to the plasma membrane, nuclear membrane and vesicular structures in response to altered Ca^2+^ concentration [22, 23]. ANXA7, via its GTPase activity, facilitates vesicle transport and membrane fusion [24]. In addition, ANXA7 may function in carcinogenesis by a discrete signaling pathway involving some tumor-suppressor, DNA-repair, and apoptosis-related genes [25, 26]. We previously found a small molecule, ABO, that could inhibit the GTPase activity of ANXA7 [27]. In the apoE^−/−^ mouse endothelium, ABO inhibited the binding of ANXA7 and ITGB4 and decreased ITGB4 Y1494 phosphorylation [28]. So ANXA7 may be involved in regulating the phosphorylation and nuclear translocation of ITGB4.

Inspired by the previous studies and given that ITGB4 is highly expressed in cancer cells, in the current study we focused on the roles of ITGB4 nuclear translocation in controlling cancer cell apoptosis. We discovered a chiral small molecule, SEC, that could specifically induce apoptosis of tumor cells with high ITGB4 content by promoting ITGB4 nuclear translocation. We provide new evidence that nuclear ITGB4 can bind to the *ATF3* promoter region and then activate transcription of downstream genes. We further demonstrated that with increased GTPase activity, ANXA7 was involved in ITGB4 nuclear translocation by increasing the phosphorylation of the ITGB4 Y1494 site. This study provides a powerful proof of concept for using the nuclear translocation of ITGB4 as a therapeutic strategy to combat various ITGB4-related cancers.

## RESULTS

### SEC specifically induces apoptosis in tumor cells with high expression of ITGB4 by promoting ITGB4 nuclear translocation

**Figure 1:**
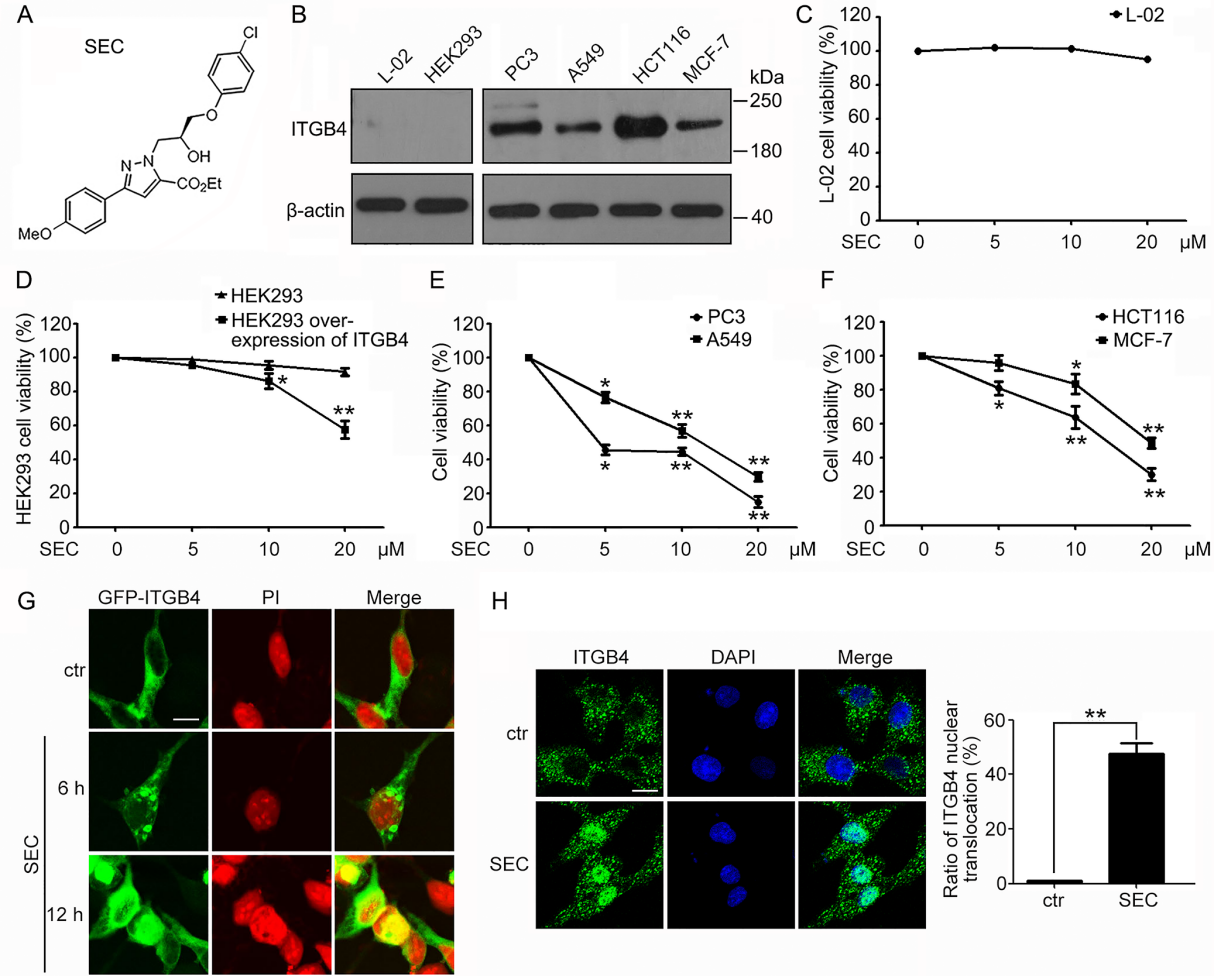
Effect of a chiral small molecule, SEC, on cell viability and ITGB4 nuclear translocation in cells with different expression of ITGB4 (**A**) The structure of SEC. (**B**) Western blot analysis of ITGB4 protein level in L-02, HEK293, PC3, A549, HCT116 and MCF-7 cells. β-actin was a loading control. Viability of (**C**) L-02 cells, (**D**) HEK293 cells and HEK293 cells with ITGB4 overexpressed, (**E**) PC3 cells and A549 cells, and (**F**) HCT116 and MCF-7 cells treated with SEC at the indicated concentrations for 24 h. (**G**) HEK293 cells were transfected with GFP-ITGB4 for 24 h, then treated with 20 μM SEC for 6 h and 12 h, and GFP-ITGB4 localization was detected by the Zeiss ZEN software. Nuclei were labeled with propidium iodide (PI). (**H**) Immunofluorescence to show the effect of SEC on ITGB4 nuclear translocation at 20 μM for 12 h in A549 cells. Nuclei were labeled with 4′,6-diamidino-2-phenylindole (DAPI). Statistical analysis shows the ratio of ITGB4 nuclear translocation. Bar, 16 μM. Data are mean ± SEM; *n* = 3; *p < 0.05; **p < 0.01.

ITGB4 was initially identified as a tumor-related antigen upregulated in multiple cancer cells [2], so investigating compounds selective for ITGB4 for cancer therapy is of interest. We used the structure of ECPC to generate an effective chiral small molecule, (*S*)-ethyl 1-(3-(4-chlorophenoxy)-2-hydroxypropyl)-3-(4-methoxyphenyl)-1H-pyrazole-5-carboxylate (SEC) (Figure 1A), that selectively inhibited growth of cells with high ITGB4 content. We examined its selectivity for inducing apoptosis in cells with differential expression of ITGB4: hepatic cells (L-02), human embryonic kidney cells (HEK293), and 4 kinds of tumor cells – prostatic cancer (PC3), lung carcinoma (A549), colorectal cancer (HCT116) and breast cancer (MCF-7) (Figure 1B). ITGB4 level was low in L-02 cells, and SEC had no effect on their viability (Figure 1C). Similarly, the viability of HEK293 cells, with low ITGB4 level, was not affected by SEC treatment (Figure 1D). However, SEC significantly decreased the viability of HEK293 cells with overexpression of ITGB4 (Figure 1D). ITGB4 is abundant in the 4 kinds of tumor cells, and SEC dose-dependently decreased the cell viability (Figure 1E and 1F). In parallel with the marked decrease in cell viability, Hoechst staining revealed that SEC augmented the apoptosis ratio of the tumor PC3, A549, HCT116 and MCF-7 cells (Supplementary Figure 1A and 1B).

Altered localization of the transmembrane receptor ITGB4 is implicated in the progression of carcinoma [3, 5, 6]. The critical roles of ITGB4 localization inspired us to detect the effect of SEC on the subcellular distribution of ITGB4. We used HEK293, which stably express GFP-ITGB4, and A549 cells, with high ITGB4 level. SEC time-dependently triggered ITGB4 nuclear translocation in GFP-ITGB4-expressing HEK293 cells (Figure 1G). The altered distribution of ITGB4 to the nucleus was also confirmed in A549 cells (Figure 1H).

### Nuclear ITGB4 regulates the transcription of target genes

**Figure 2:**
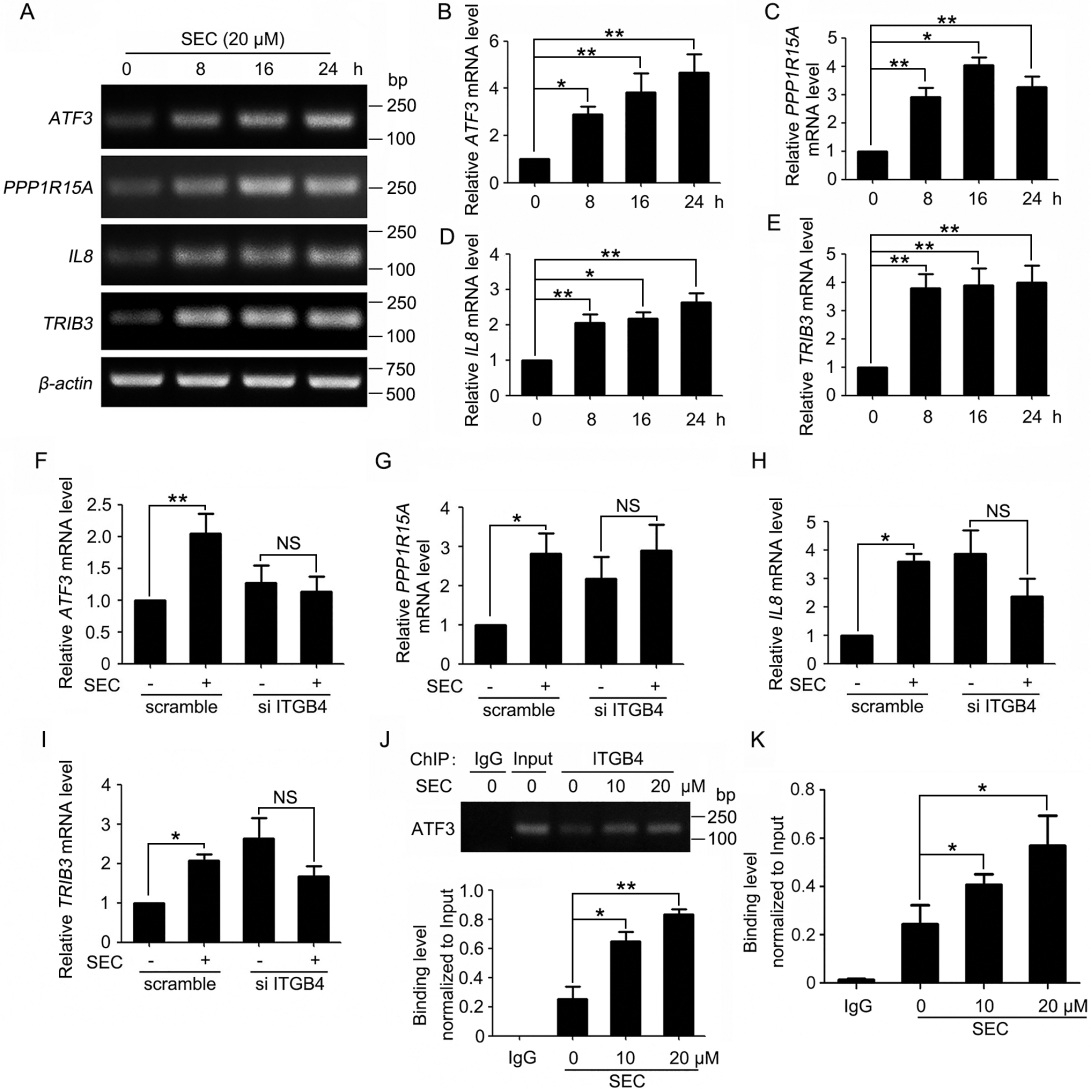
Activation of gene expression by nuclear ITGB4 (**A**) RT-PCR analysis of mRNA levels of *ATF3*, *PPP1R15A*, *IL8* and *TRIB3* treated with SEC (20 μM) for indicated times. (**B**, **C**, **D** and **E**) Quantified bands of Figure 2A using ImageJ. mRNA levels were normalized to that of *β-actin*. (**F**, **G**, **H** and **I**) qPCR analysis of mRNA levels of *ATF3*, *PPP1R15A*, *IL8* and *TRIB3* treated with SEC (20 μM) for 24 h with or without ITGB4 siRNA. (J and K) Effects of SEC treatment on the binding of ITGB4 to the *ATF3* promoter. PC3 cells treated with SEC were crosslinked, fractionated, and submitted to (**J**) ChIP-PCR and (**K**) ChIP-qPCR analysis. Band density was quantified by using ImageJ. Data are mean ± SEM; *n* = 3; *p < 0.05; **p < 0.01; NS, no significance.

The nuclear redistribution of ITGB4 prompted us to search for potential target genes that might be regulated by nuclear ITGB4. Therefore, we performed microarray assay to analyze the gene expression profile with ITGB4 nuclear translocation triggered by SEC. Microarray assay revealed increased expression of a number of apoptosis-related genes. We selected the most upregulated genes, *CITED2*, *PPP1R15A*, *IL*8, *ATF3* and *TRIB3* (Table 1), for further investigation. Oligonucleotide primers for the genes of interest were designed (Supplementary Table 1). The mRNA levels of *PPP1R15A*, *IL8*, *ATF3* and *TRIB3* were indeed increased with SEC stimulation (Figure 2A–2E), with negligible effect on *CITED2* transcription (Supplementary Figure 2A). After RNAi-mediated knockdown of ITGB4, SEC stimulation had no effect on the expression of target genes (Figure 2F–2I and Supplementary Figure 2B).

**Table 1:** Microarray analysis shows the five most upregulated genes

Gene symbol	Fold change (SEC VS ctr)	Gene name
*CITED2*	9.4115	Cbp/p300-interacting transactivator 2
*PPP1R15A*	9.0298	Protein phosphatase 1, regulatory subunit 15A
*IL8*	8.4076	Interleukin-8 precursor
*ATF3*	7.71255	Activating transcription factor 3
*TRIB3*	7.1499	Tribbles homolog 3

*ATF3* is needed for full induction of *PPP1R15A* expression [29]. Loss of *ATF3* function blocked the transcription of *IL8* [30]. Increased *ATF3* level is accompanied by the upregulation of *TRIB3* during apoptosis [31, 32]. Therefore, nuclear ITGB4 might promote apoptosis by binding to the *ATF3* promoter region, thereby promoting the expression of *ATF3* and upregulating downstream apoptosis-related genes. To test this hypothesis, we predicted 8 binding sites 2-kb upstream of the *ATF3* promoter region and performed chromatin immunoprecipitation (ChIP) to detect ITGB4 occupancy at each of the 8 putative regions with primers specific for the predicted regions (Supplementary Table 2). Consistently, semiquantitative RT-PCR and quantitative RT-PCR (qPCR) confirmed that SEC activated the recruitment of ITGB4 to the sixth predicted binding site (Figure 2J and 2K), with no binding ability with the other 7 sites (Supplementary Figure 2C). These results indicate the binding of ITGB4 to the promoter of *ATF3* in the upregulation of *ATF3* and the downstream gene transcription.

### ANXA7 is involved in ITGB4 nuclear translocation

**Figure 3:**
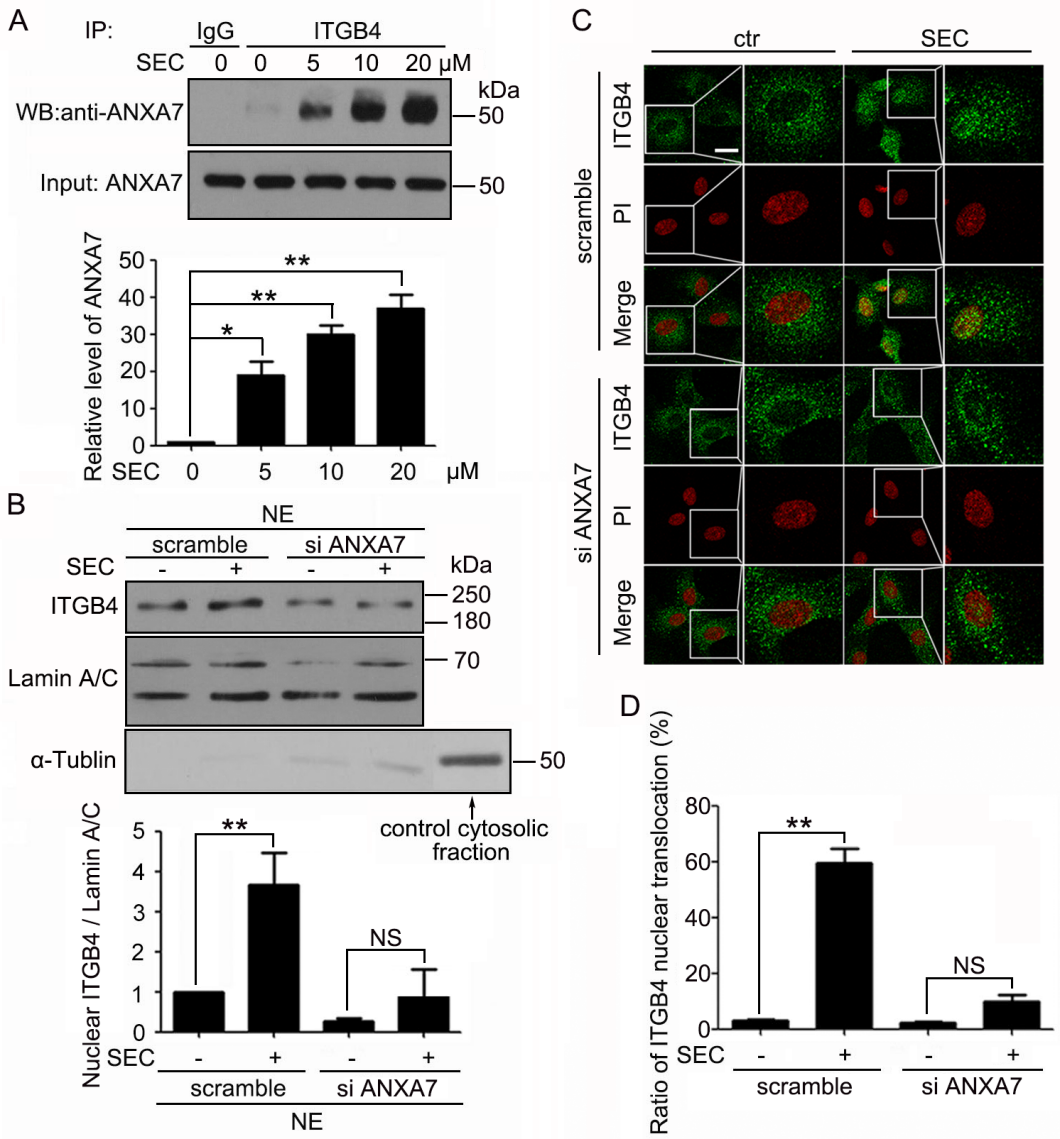
ANXA7 binds to ITGB4 and is required for ITGB4 nuclear translocation (**A**) Western blot (WB) analysis of co-immunoprecipitation (co-IP) of ANXA7 with ITGB4 antibody in PC3 cells treated with SEC at the indicated concentrations with 1% fetal bovine serum (FBS) for 24 h and quantification of ANXA7 level. (**B**) Subcellular fractionation of PC3 cells to detect nuclear ITGB4 level after treatment with SEC at 20 μM for 24 h with and without siRNA ANXA7. WB analysis of Lamin A/C and α-Tubulin to confirm the specificity of the cell fractionation protocol. The nuclear marker Lamin A/C was a loading control. (**C** and **D**) Immunofluorescence analysis of localization of ITGB4 in A549 cells transfected with and without ANXA7 siRNA for 24 h, then treated with 20 μM SEC for 12 h with 1% FBS, and the percentage of cells containing nuclear ITGB4. Nuclei were labeled with PI. Bar, 16 μM. Data are mean ± SEM; *n* = 3; *p < 0.05; **p < 0.01; NS, no significance.

To illuminate the mechanism by which ITGB4 translocated to the nucleus, we investigated the key regulatory factors. We performed co-immunoprecipitation assay with PC3 cells and found that SEC dose-dependently promoted the binding of ANXA7 to ITGB4 (Figure 3A). Therefore, ANXA7 may participate in the nuclear translocation of ITGB4.

To test this hypothesis, we performed biochemical fractionation with PC3 cells after treatment with ANXA7 siRNA for 24 h followed by SEC stimulation for 24 h. The efficiency of ANXA7 knockdown was analyzed by western blot analysis (Supplementary Figure 3A), which was concomitant with minor levels of nuclear ITGB4 (Figure 3B). As well, on immunofluorescence analysis, treatment with ANXA7 siRNA blocked the capacity of SEC to induce nuclear localization of ITGB4 (Figure 3C, 3D and Supplementary Figure 3B). This finding suggests that ANXA7 is responsible for ITGB4 nuclear translocation.

### ANXA7 promotes ITGB4 nuclear translocation by exerting GTPase activity

**Figure 4:**
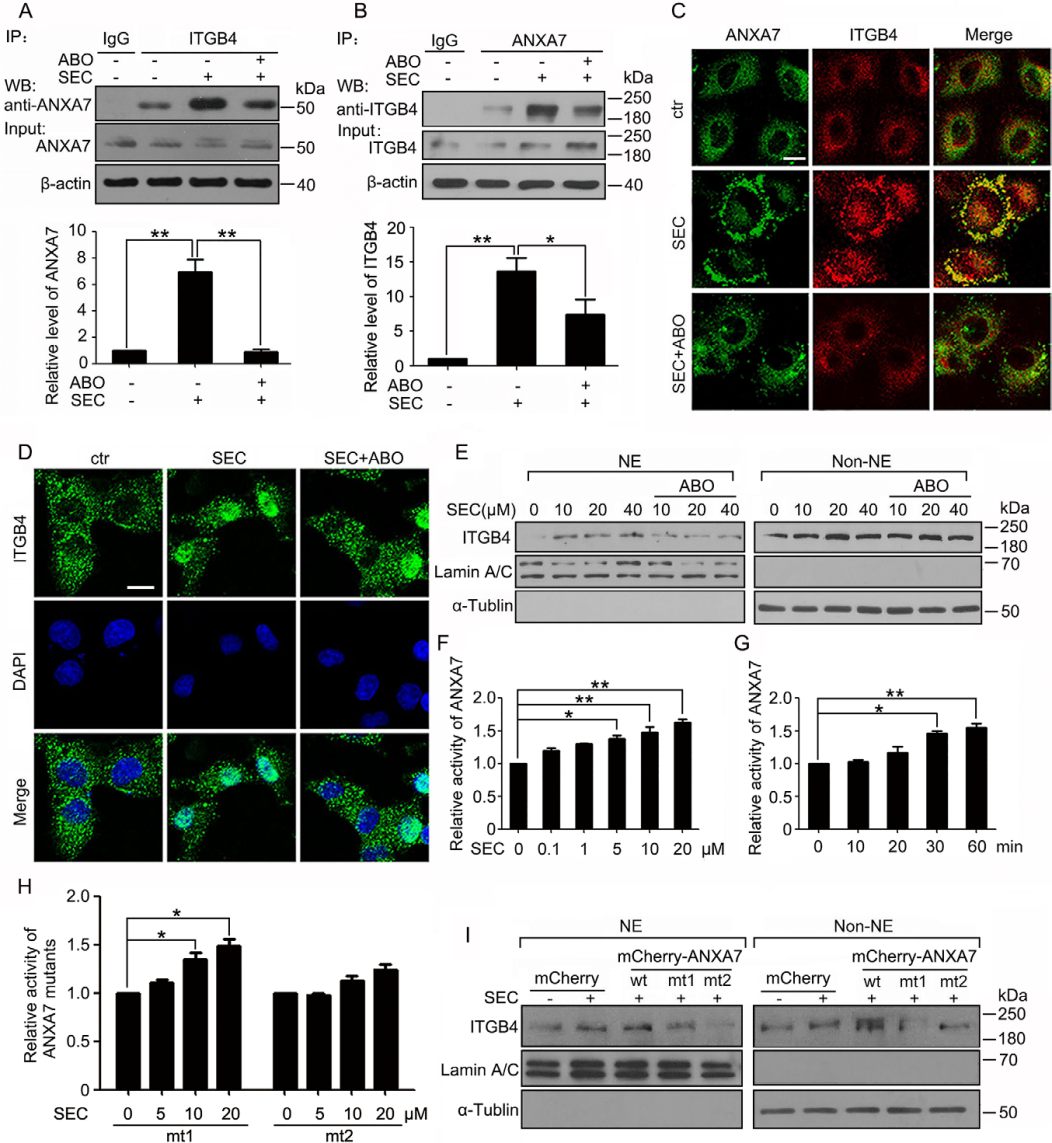
ANXA7 GTPase activity promotes ITGB4 nuclear translocation WB analysis of co-IP of (**A**) ANXA7 with ITGB4 antibody and (**B**) ITGB4 with ANXA7 antibody in PC3 cells treated with SEC (20 μM) with or without ABO (50 μM) with 1% FBS for 24 h and quantification of co-immunoprecipitated ANXA7 and ITGB4 levels. (**C**) Immunofluorescence assay of the co-localization of ANXA7 and ITGB4 treated with SEC (20 μM) with or without ABO (50 μM) for 6 h. (**D**) Immunofluorescence of ITGB4 in A549 cells incubated with SEC (20 μM) with or without ABO (50 μM) for 12 h. Nuclei were labeled with DAPI. (**E**) WB analysis of nuclear translocation of ITGB4 after subnuclear fractionation of PC3 cells treated with SEC at the indicated concentrations with or without ABO (50 μM) for 24 h. (**F** and **G**) Dose-dependent effect of SEC on ANXA7 GTPase activity with incubation for 60 min, and time-dependent effect at 20 μM. (**H**) Effect of SEC on ANXA7-mt1 (T275A) and -mt2 (T286A) GTPase activity at the indicated concentrations for 60 min. (**I**) WB analysis of localization of nuclear and cytoplasmic ITGB4 in PC3 cells transfected with expression vectors mCherry and mCherry-ANXA7-wt, -mt1 (T275A) and -mt2 (T286A) for 48 h, then treated with 20 μM SEC for 24 h with 1% FBS. NE and Non-NE, nucleus and non-nucleus components, respectively. Bar, 16 μM. Data are mean ± SEM; *n* = 3; *p < 0.05; **p < 0.01.

Given that ANXA7 possesses GTPase property, we wondered whether ANXA7 contributed to ITGB4 redistribution by exerting its GTPase activity. ABO inhibits ANXA7 GTPase activity by decreasing phosphorylation at ANXA7 Thr286 with no effect on phosphorylation at Thr275 [27]. We found that ABO inhibited the SEC-promoted binding of ANXA7 and ITGB4 (Figure 4A and 4B). SEC stimulated the co-precipitation of ITGB4 with mCherry-ANXA7-wt (wild type) and -mt1 (T275A) (mutant) but not non-phosphorylatable mCherry-ANXA7-mt2 (T286A). mCherry-ANXA7-mt3 (T286D), a phospho-mimic mutant, facilitated the binding of ITGB4 and ANXA7 (Supplementary Figure 4A). In agreement, immunofluorescence assay revealed that SEC facilitated the co-localization of the 2 proteins, and ABO effectively reversed the phenomenon (Figure 4C and Supplementary Figure 4B). Next, we explored the function of ANXA7 GTPase in ITGB4 nuclear translocation. On immunofluorescence and subcellular fractionation assay, SEC-triggered ITGB4 nuclear translocation was compromised with ABO treatment (Figure 4D, 4E and Supplementary Figure 4C).

In addition, as ITGB4 nuclear translocation was suppressed by ABO, the decreased PC3 cell viability induced by SEC was attenuated (Supplementary Figure 4D). With ABO treatment, the SEC-increased *ATF3* gene expression was compromised, accompanied by decreased expression of *PPP1R15A*, *IL8* and *TRIB3* (Supplementary Figure 4E).

For the vital importance of ANXA7 GTPase activity in the stimulation of ITGB4 nuclear trafficking, we evaluated the effect of SEC on ANXA7 GTPase activity. We transfected HEK293 cells with plasmids encoding 6his-ANXA7 and 6his-ANXA7-mt1 (T275A) and -mt2 (T286A) mutants for 48 h. SEC dose- and time-dependently increased the activity of 6his-ANXA7 purified from HEK293 cells (Figure 4F and 4G). The point mutation to alanine at Thr275 had no effect on SEC-increased ANXA7 activity (Figure 4H and Supplementary Figure 5A). In contrast, with mutation of Thr286 to alanine, SEC could not increase ANXA7 GTPase activity (Figure 4H and Supplementary Figure 5A).

After transfecting HEK293 cells with plasmids coding for mCherry-ANXA7-wt, -mt1 (T275A) and -mt2 (T286A) and GFP-ITGB4 for 48 h, we used antibodies specific for ANXA7 and ITGB4 to immunoprecipitate ANXA7 and ITGB4 fusion proteins, respectively, and examined whether SEC interfered in the process. SEC dose-dependently blocked the binding of mCherry-ANXA7-wt and -mt1 (T275A) to their antibodies (Supplementary Figure 5B and 5C) but had no effect on the binding of mCherry-ANXA7-mt2 (T286A) with its antibody (Supplementary Figure 5D). Moreover, SEC did not interfere with the binding of GFP-ITGB4 to its antibody (Supplementary Figure 5E). When we immunoprecipitated mCherry-ANXA7-wt with mCherry antibody, the level of immunoprecipitated mCherry-ANXA7-wt was not changed with SEC treatment (Supplementary Figure 5F), and the level of immunoprecipitated GFP-ITGB4 with GFP antibody was not changed (Supplementary Figure 5G). Therefore, SEC selectively and directly bound to ANXA7 but not ITGB4. Thr286 in ANXA7 was a key binding site. SEC increased ANXA7 GTPase activity by binding to ANXA7 at Thr286.

Subcellular fractionation analysis revealed that PC3 cells transfected with mCherry-ANXA7-mt2 (T286A) expressed extreme low levels of nuclear ITGB4 as compared with cells transfected with mCherry-ANXA7-wt and -mt1 (T275A) (Figure 4I). These results reinforced the importance of ANXA7 GTPase activity in controlling ITGB4 nuclear translocation.

### ANXA7 GTPase activity enhances ITGB4 Y1494 phosphorylation and its subsequent nuclear translocation

**Figure 5:**
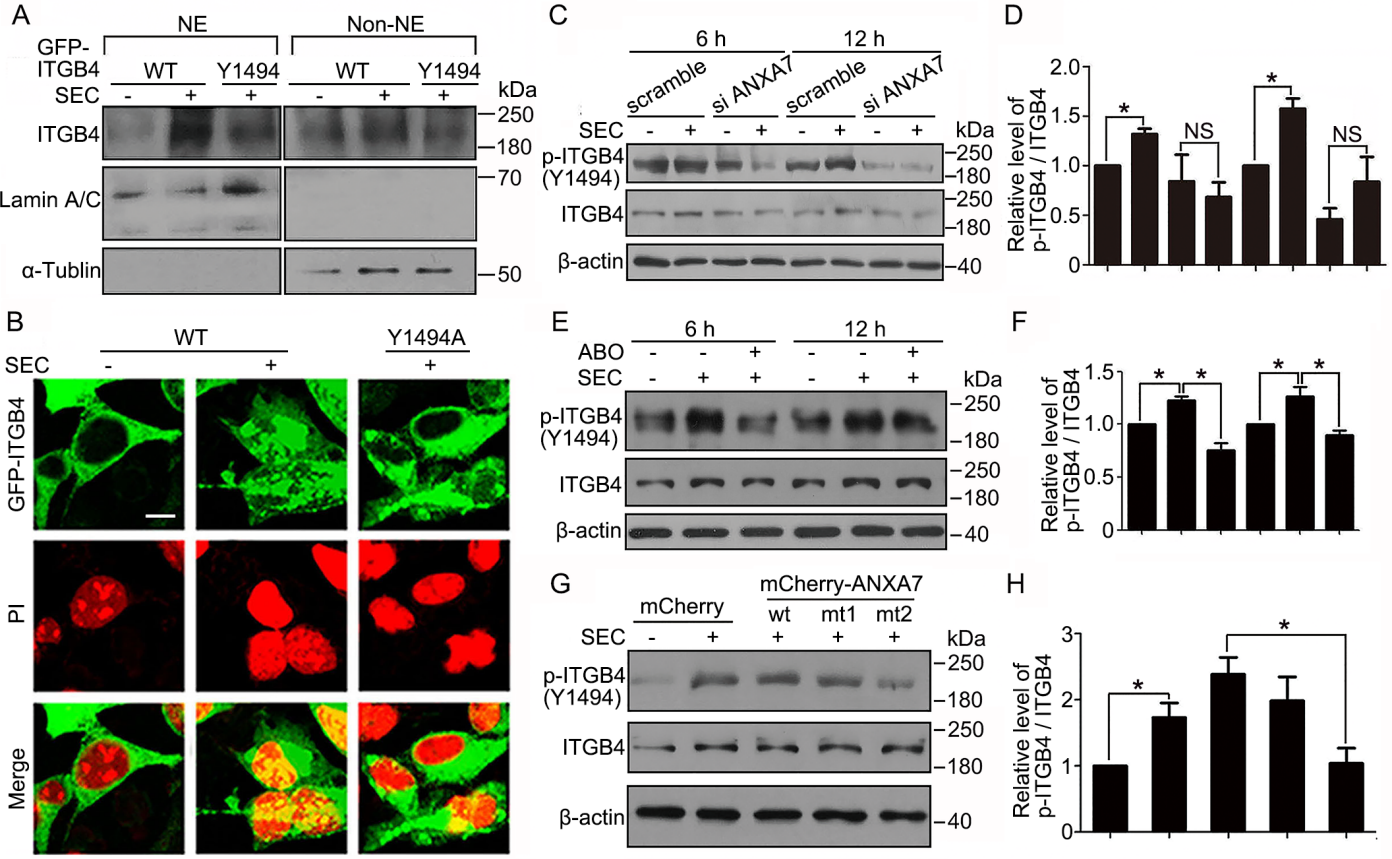
Y1494 phosphorylation of ITGB4 by ANXA7 is responsible for its nuclear translocation Transfection of HEK293 cells with GFP-ITGB4 (WT) or GFP-ITGB4-mt (Y1494A) for 24 h, followed by 20 μM SEC treatment for 24 h. (**A**) WB analysis of nuclear translocation of ITGB4 after subnuclear fractionation. NE and Non-NE, nucleus and non-nucleus components, respectively. (**B**) Immunofluorescence of ITGB4 nuclear translocation. Nuclei were labeled with PI. WB analysis of phosphorylated ITGB4 at Y1494 and total ITGB4 in PC3 cells treated with SEC (20 μM) (**C** and **D**) with or without siRNA ANXA7 and (**E** and **F**) with or without ABO (50 μM) for the indicated times and (**G** and **H**) with expression vectors mCherry and mCherry-ANXA7-wt, -mt1 (T275A) and -mt2 (T286A) for 12 h, and the quantification of phosphorylated ITGB4 at Y1494. Bar, 16 μM. Data are mean ± SEM; *n* = 3; *p < 0.05; NS, no significance.

Tyr1494 in ITGB4 functions as a master regulator controlling multiple signal transduction pathways closely related to tumor progression [15, 19, 20]. To detect the role of Y1494 in ITGB4 nuclear trafficking, we transfected HEK 293 cells with wild-type ITGB4 and ITGB4 containing a point mutation to alanine at Y1494. ITGB4 nuclear localization was reduced more in cells with expression of the Y1494A mutant than wild-type ITGB4 (Figure 5A). On immunofluorescence assay, SEC clearly induced nuclear translocation of the wild-type ITGB4 but had little effect on the Y1494A mutant (Figure 5B).

To confirm the contribution of ANXA7 in regulating the phosphorylation of ITGB4, we transfected ANXA7 siRNA into PC3 cells and showed that Y1494 phosphorylation of ITGB4 was markedly inhibited by blocking ANXA7 expression (Figure 5C and 5D). Because SEC increased and ABO decreased ANXA7 GTPase activity, SEC elevated the phosphorylation status at ITGB4 Y1494, which was inhibited by ABO (Figure 5E and 5F). In addition, phosphorylation of ITGB4 Y1494 was lower in PC3 cells transfected with mCherry-ANXA7-mt2 (T286A) than mCherry-ANXA7-wt (Figure 5G and x5H). Thus, by exerting GTPase activity, ANXA7 promoted ITGB4 Y1494 phosphorylation and its subsequent nuclear translocation induced by SEC.

### SEC inhibited the growth of human tumor xenografts in an avian embryo model

**Figure 6:**
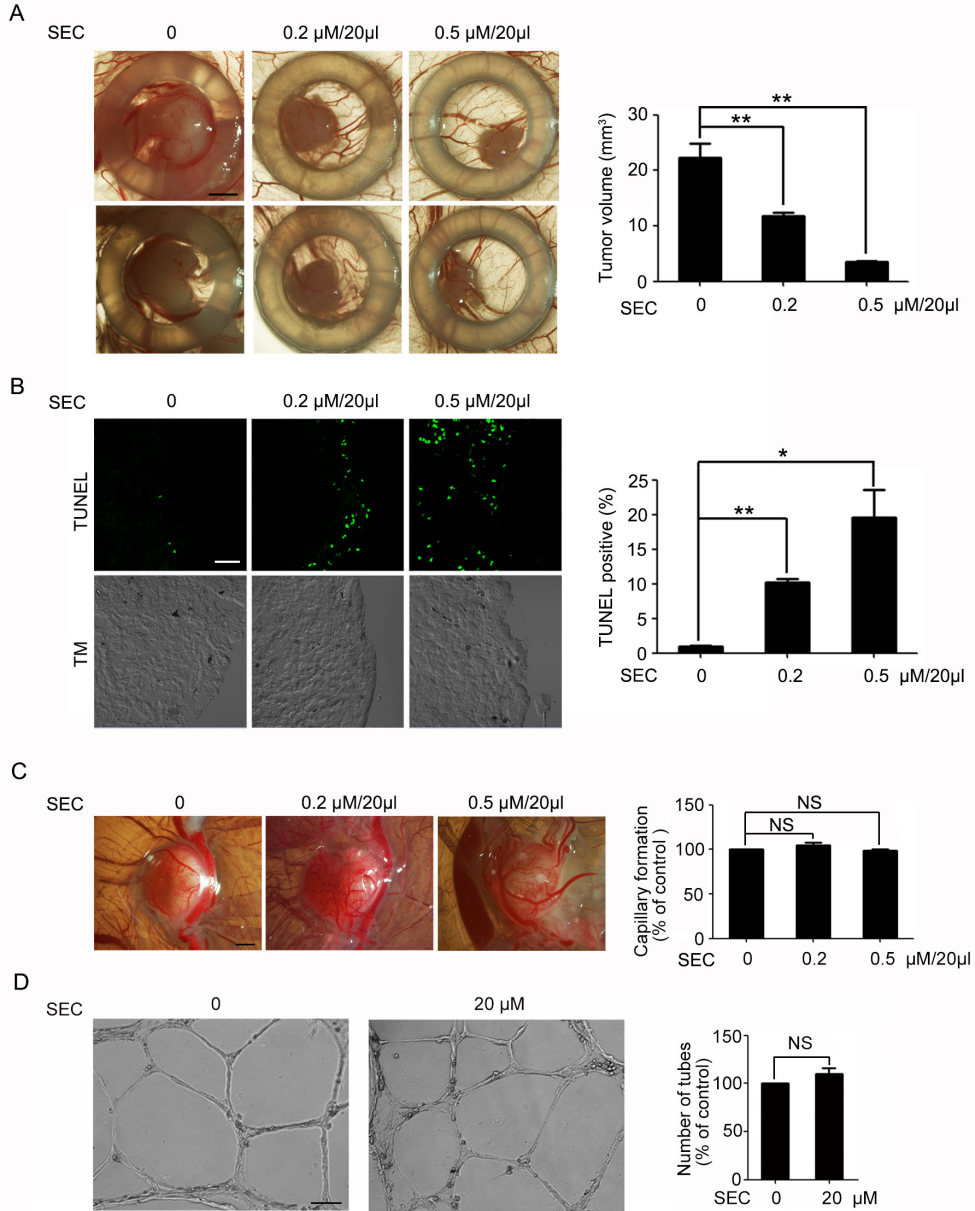
Topical treatment of experimental tumor with SEC (**A**) Biomicroscopy imaging of control and treated tumors. Tumor volumes were quantified. Control group show progressive growth, whereas SEC-treated tumors show smaller tumor volume. Bar, 1.5 mm. *n* = 5. (**B**) TUNEL staining and confocal microscopy analysis of the frozen sections of Day 8 experimental tumors. Apoptotic rate with and without SEC treated was quantified by TUNEL staining. Bar, 32 μM. *n* = 5. (**C**) Biomicroscopy and quantification of angiogenesis on gelatin sponge with and without SEC adsorption. SEC had no effect on capillary formation. Bar, 2 mm. *n* = 5. (**D**) Inverted-phase contrast microscopy to show the effects of SEC on formation of the vascular-like structures in VECs on Matrigel, and its quantification indicated that the tube formation was not affected by SEC. Bar, 100 μM. *n* = 3. Data are mean ± SEM; *p < 0.05; **p < 0.01. NS, no significance.

Next, we determined whether SEC had apoptosis-promoting effects *in vivo*. Because of the immune-deficient environment and the dense capillary network, the chick embryo chorioallantoic membrane (CAM) is widely used for tumor engraftment to evaluate the efficacy of anticancer drugs [33, 34] and for the action of proangiogenic or antiangiogenic factors [35]. Therefore, we used the CAM model to study the action of SEC in tumor growth as well as normal angiogenesis. A549 cells were deposited on the CAM and formed a solid tumor within 2 days. The solid adenocarcinoma was locally treated from day 2 to 8 with PBS or PBS containing SEC every 2 days. Treatment with PBS plus SEC substantially inhibited tumor growth as compared with PBS alone (Figure 6A). 5FU treatment was beneficial in reducing the size of xenografts (Supplementary Figure 6). TUNEL assay of frozen sections of tumors revealed that SEC effectively promoted apoptosis *in vivo*, and an even stronger signal was detected in the peritumoral edge (Figure 6B). To determine whether SEC disturbed normal angiogenesis accompanied by suppressing tumor growth, we directly measured the angiogenic action of SEC on CAM. SEC had no effect on CAM normal angiogenesis (Figure 6C). Fully consistent with the *in vivo* angiogenesis studies, SEC did not interfere with the formation of capillary-like tube structures with serum and FGF-2 *in vitro* Matrigel assay (Figure 6D). Therefore, SEC effectively inhibited tumor growth *in vivo* by inducing apoptosis with no adverse effects on normal CAM angiogenesis.

## DISCUSSION

In this study, we identified a new chiral small molecule, SEC, that could promote cancer cell apoptosis by inducing ITGB4 nuclear translocation. We provide the novel function of ITGB4 in the nucleus by binding to the *ATF3* promoter and demonstrate the regulatory roles of ANXA7 in ITGB4 nuclear trafficking by promoting the phosphorylation of ITGB4 Y1494.

ITGB4 is highly expressed in multiple tumors, such as lung carcinoma, prostate cancer, colorectal cancer and breast cancer [2], and is implicated in clinical investigation [36]. Most studies have focused on the involvement of ITGB4 in tumor proliferation, invasion and metastasis. Here, we highlight the physiological significance of ITGB4 nuclear translocation in promoting tumor cell apoptosis. Unlike blood vessels delivering abundant nutrients to healthy tissues, tumor vasculature is tortuous and leaky, functions poorly and results in a nutrient-limited hostile tumor microenvironment [37, 38]. In this study, we investigated the action of SEC in a more physiological microenvironment with low serum in tumor cells and appropriate serum in normal cells. Strikingly, SEC specifically promoted apoptosis by inducing ITGB4 nuclear translocation in a tumor-specific manner, with no side effects in normal cells.

All of the genes upregulated in response to SEC have potential links with apoptosis. *ATF3* has low expression in normal cells and upregulated with stress signals involved in apoptosis [39–41]. Accumulating studies indicate that *ATF3* may work as a master regulator controlling the expression of *PPP1R15A* [29, 42], *IL8* [30] and *TRIB3* [31, 32, 43, 44]. Our ChIP results showed that nuclear ITGB4 bound to the promoter region of *ATF3*. The significant and specific SEC-induced binding of ITGB4 to the *ATF3* promoter activates *ATF3* transcription and explains the increased expression of the downstream genes *PPP1R15A*, *IL-8* and *TRIB3*. Hence, we provide the novel roles of nuclear ITGB4 in stimulating the transcription of target genes.

Use of the PSORT II prediction program (http://psort.hgc.jp/form2.html) revealed that ITGB4 possesses 2 putative polybasic nuclear localization signals (NLSs) — aa 700-HKKK-705 and 1662-RPRR-1667. Emerging evidence has shown that transmembrane proteins, such as receptor tyrosine kinases (RTKs) and p75 neurotrophic receptor, can translocate from the cell membrane to the nucleus [45–48]. EGFR and ErbB2 migrate to the nucleus by endocytosis and binding to importin β1 via NLSs. The activation of dynamin and Ran GTPases is required for endocytic internalization and trafficking [49, 50]. ANXA7, via its GTPase activity, facilitates membrane vesicle fusion during endocytosis and exocytosis [24, 51, 52]. Here we confirmed that SEC-activated ANXA7 GTPase was responsible for promoting ITGB4 nuclear transport.

The phosphorylation of ITGB4 cytoplasmic domain could contribute to the changes in ITGB4 localization. PKC-mediated phosphorylation of S1356, S1360 and S1364 leads to HD disruption and ITGB4 mobilization to lamellipodia [3, 17]. ITGB4 phosphorylation at S1354 and S1362 causes ITGB4 endocytosis via Rab5 and Rab11 compartments [53]. Moreover, EGFR nuclear transport is initiated by the phosphorylation of Y1101 and S229 [54, 55]. Here, we revealed that ANXA7 activation resulted in Y1494 phosphorylation of ITGB4, which triggered ITGB4 redistribution to the nucleus. The activated GTPase family is widely implicated in promoting target phosphorylation via intermediate kinases. Rac1 GTPase activation promotes phosphorylation of myosin II heavy chain on Ser1916 in a PKC-dependent mechanism that enhances cell migration [56]. Rho-associated protein kinase (ROCK) is an effector of the small GTPase family Rho. Activation of Rho A and B small GTPases induced by transforming growth factor β1 (TGF-β1) increases LIM-kinase 2 (LIMK2) phosphorylation by ROCK. Activated LIMK2 then phosphorylates cofilin to promote actin reorganization [57]. We previously reported that ABO-induced inhibition of ANXA7 GTPase activity decreased the phosphorylation of GCA and TIA-1 [27, 58]. In the current study, SEC-activated ANXA7 GTPase increased the phosphorylation of ITGB4. Therefore, possible ANXA7 GTPase downstream kinase effectors may regulate the phosphorylation of target proteins. So far, the kinase effectors of ANXA7 GTPase have not been reported. Both Met and FGFR1 can promote ITGB4 Y1494 phosphorylation [15, 59]. SFKs phosphorylate major tyrosines located in the ITGB4 cytoplasm domain [2]. However, the receptor tyrosine kinases or cytoplasmic kinases are prone to bind to ITGB4 and form distinct complexes by recruiting other molecules [60–62]. Therefore, we presumed that activated ANXA7 associated with the related kinases to promote ITGB4 phosphorylation. ANXA7 indirectly associated with ITGB4 by capturing potential kinases, which acted as a linker to combine ITGB4 and ANXA7 to form a complex. The explicit mechanism needs to be explored.

Both SEC and ECPC can promote ITGB4 phosphorylation on Y1494, the ensuing ITGB4 nuclear translocation and the upregulation of apoptosis-related genes. The two small molecules function in a distinct action mechanism in different cell types. ECPC-induced ITGB4 phosphorylation was promoted by FGFR1 in vascular endothelium cells. SEC enhanced ITGB4 phosphorylation by binding to ANXA7 and activating ANXA7 GTPase in tumor cells. Whether a cross-link exists between the two mechanisms remains to be demonstrated. In addition, we extended our previous study by shedding light on the novel function of ITGB4 in the nucleus by binding to the *ATF3* promoter. The novel role of nuclear ITGB4 helps to explain the upregulation of apoptotic genes stimulated by ECPC and SEC.

**Figure 7:**
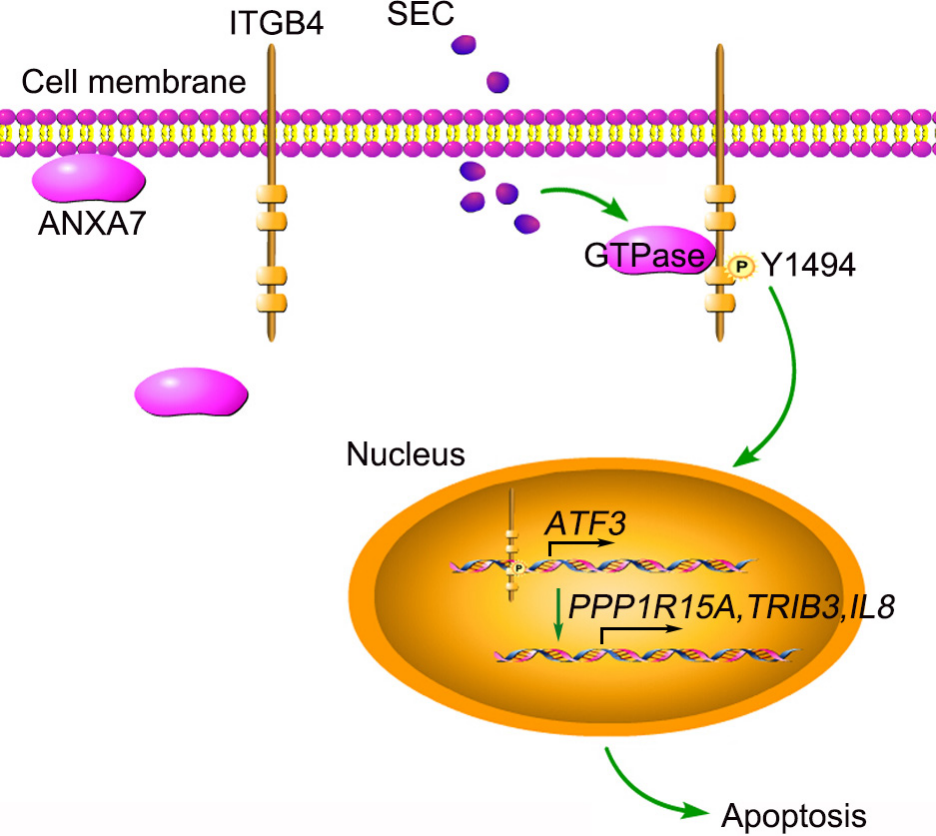
Schematic presentation of SEC-induced apoptosis by triggering ITGB4 nuclear translocation SEC treatment increases ANXA7 GTPase activity and promotes the binding of ANXA7 to ITGB4, which contributes to the phosphorylation of ITGB4 Y1494. Subsequently, ITGB4 translocates to the nucleus. Nuclear ITGB4 binds to the *ATF3* promoter region, activates *ATF3* transcription and promotes the expression of downstream apoptosis-related genes.

In summary, we found that a small molecule, SEC, selectively promoted apoptosis in tumor cells with high expression of ITGB4. After enhancing ANXA7 GTPase activity, SEC triggers ITGB4 phosphorylation at Y1494, which induces ITGB4 nuclear translocation. Nuclear ITGB4 binds to *ATF3* promoter regions and promotes the transcription of downstream genes related to apoptosis (Figure 7). These results extend the scope of our study, emphasizing the roles of ITGB4 nuclear translocation in controlling tumor cell apoptosis and the explicit mechanism of pro-apoptosis signaling. The specificity of SEC for nuclear trafficking of ITGB4 and the apoptosis-promoting effect support potential ITGB4 target drugs to combat ITGB4-related tumors.

## MATERIALS AND METHODS

### Synthesis of the compound SEC

(*S*)-2-((4-chlorophenoxy)methyl)oxirane was synthesized by the reaction of 4-chlorophenol with (*S*)-oxiran-2-ylmethyl 4-methylbenzenesulfonate. (*S*)-ethyl 1-(3-(4-chlorophenoxy)-2-hydroxypropyl)-3-(4-methoxyphenyl)-1H-pyrazole-5-carboxylate (SEC) was obtained by the reaction of ethyl 3-(4-methoxyphenyl)-1H-pyrazole-5-carboxylate with (*S*)-2-((4-chlorophenoxy) methyl)oxirane according to our previous report [63].

### Antibodies

Antibodies for integrin β4 (sc-9090), ANXA7 (sc-11389), α-Tubulin (sc-5286), β-actin (sc-47778), GFP (sc-9996) and horseradish peroxidase-conjugated secondary antibodies were from Santa Cruz Biotechnology. Lamin A/C (2032) antibody was from CST. Antibody for phospho-Y-1494 integrin β4 (ab29043) and mCherry (ab167453) were from Abcam. Secondary antibodies for immunofluorescence were goat anti-mouse IgG Alexa Fluor-488 (A11029) and goat anti-rabbit IgG Alexa Fluor-549 (A-11037; both Invitrogen).

### Cell culture

HEK293 cells and MCF-7 cells were grown in Dulbecco's modified Eagle's medium (DMEM, Gibco, 12800–058) with 10% fetal bovine serum (FBS; Hyclone, SV30087.02). L-02, PC3, A549 and HCT116 cells were cultured in RPMI Medium 1640 (Gibco, 3180–022) in the presence of 10% FBS (Hyclone, SV30087.02). All cell lines were cultured in a humidified incubator at 37°C with 5% CO_2_.

### Cell viability assay

Treated cells were plated in 96-well plates, then precipitated for 1 h at 4°C with 100 μl 10% trichloroacetic acid (Shenggong Biotech, Shanghai) and stained with 50 μl sulforhodamine B (SRB; Sigma-Aldrich, USA). The optical density was read at 540 nm after reconstitution of the bound dye in 100 μl of 10 mM Trisbase (pH 10.5) by use of a Spec-traMAX 190 microplate spectrophotometer (GMI Co., USA). Cell viability (%) = (OD of treated group/OD of control group) × 100.

### Hoechst 33258 staining

Live PC3, A549, HCT116 and MCF-7 cells were stained with Hoechst 33258 at 10 mg/mL in the medium for 15 min at 37°C. Cells washed with PBS twice were observed under a fluorescence microscope (Nikon). Apoptotic cells were identified by intense local staining of condensed DNA, with diffuse DNA staining in normal cells. At least 400 cells were counted for apoptotic cells.

### Cell morphology

Morphologic changes of PC3 cells were observed by inverted phase-contrast microscopy (Eclipse TS-100; Nikon, Tokyo).

### Immunofluorescence assay

Treated cells were fixed in 4% paraformaldehyde (w/v) for 30 min at room temperature, then incubated with normal goat serum (1:30) for 20 min and primary antibodies (1:100) overnight at 4°C. Cells were washed with phosphate buffered saline (PBS) 3 times, then incubated with secondary antibodies (1:200) for 1 h at 37°C. Fluorescence was detected by laser scanning confocal microscopy (Leica, Wetzlar, Germany).

### RT-PCR and quantitative real-time PCR

Extraction of total RNA involved use of Trizol reagent (Invitrogen, USA). cDNA was synthesized with 2 μg RNA by use of M-MLV reverse transcriptase (Promega, USA). For RT-PCR, mastermix (MegaMix, Cambio) was used. For quantitative real-time PCR, the ABI7000 system with SYBR Green PCR master Mix (Takara, DRR041A) was used. The expression of β-actin was used to normalize with a melting curve for each reaction. Primers for PCR were for ITGB4, sense 5′-TCTGGCCTTCAATGTCGTCT-3′, and anti-sense 5′-GGGATGATGGGGATGGACAT-3′; and β-actin, sense 5′-GAAGTGTGACGTGGACATCC-3′, and anti-sense 5′-CCGATCCACACGGAGTACTT-3′. Primers for microarray confirmation are in Supplementary Table 1.

### Chromatin immunoprecipitation (ChIP)

ChIP was performed with ChIP kits (Millipore, 17–10086) according to the user manual. Briefly, PC3 cells were fixed in 1% formaldehyde incubated at room temperature for 10 min. Approximately 10^7^ cells were lysed in 0.5 ml lysis buffer. Isolated chromatin was sheared by sonication to a mean length of about 500 bp. An amount of 50 μl of chromatin was immunoprecipitated with 6 μg ITGB4 antibody and 1 μg normal rabbit IgG for negative control overnight at 4°C with rotation; 20 μl fully resuspended protein A/G magnetic beads were added. The Protein A/G bead-antibody/chromatin complexes were washed and eluted. Then the cross-links of protein/DNA complexes were reversed to free DNA. Finally, 50 μl DNA solution was obtained for further analysis. Eight primers for PCR are in Supplementary Table 2.

### Terminal deoxynucleotidyl transferase-mediated dUTP nick-end labeling (TUNEL)

The TUNEL assay was performed as described [64]. The DeadEndTM Fluorometric TUNEL System (Promega, USA) was used to detect DNA fragmentation of treated cells and tumor tissues. Apoptosis was assessed by laser scanning confocal microscopy (Leica, Wetzlar, Germany).

### *In vitro* ANXA7 activity assay

The coding region of human wide type ANXA7 and ANXA7-mt1 (T275A) and -mt2 (T286A) mutants were subcloned into the mCherry-N1 expression vector with a 6*His tag. When the density of HEK293 plated onto 10-cm dishes reached 70% to 80% confluence, cells were transfected with the indicated expression vector. At 24 h after transection, total proteins were harvested, and expressed recombinant ANXA7 was extracted and purified by column chromatography by use of the His GraviTrap Ni-NTA protein purification assay kit. Purified ANXA7 was incubated at 37°C with or without SEC for indicated concentrations and times. The GTPase activity of ANXA7 was measured with use of the ATPase/GTPase Activity Assay Kit (MAK113, Sigma, USA).

### Western blot analysis

Western blot assay was conducted as previously described [65]. Equal amounts of protein were applied to 9% SDS-polyacrylamide gel for ITGB4 and 12% SDS-polyacrylamide gel for other proteins. Proteins in gels were electroblotted onto poly-vinylidene difluoride membranes and blocked at room temperature for 1 h, then probed with primary antibodies overnight at 4°C. After 3 washes in PBST, membranes were incubated with peroxidase-conjugated secondary antibodies for 1 h at room temperature, and proteins were detected by use of an enhanced chemiluminesence detection kit (Thermo Fisher, 34080). The relative quantity of protein levels was analyzed by use of ImageJ and normalized to loading controls.

### Immunoprecipitation (IP)

PC3 cells were lysed in IP buffer (Beyotime, P0013). The lysates were pre-cleared with protein A/G agarose beads (Beyotime, P2012) for 1 h at 4°C. After centrifugation, the supernatant was collected and incubated with specific antibodies or normal corresponding IgG, then with protein A/G beads overnight at 4°C. The beads were rinsed with IP buffer 3 times and eluted with 2 × SDS loading buffer. The immunoprecipitated proteins were detected by western blot assay.

### Plasmids and overexpression

mCherry-labelled ANXA7 wild-type (mCherry-ANXA7-wt) and mCherry-ANXA7-mt1 (T275A), -mt2 (T286A) and -mt3 (T286D) mutants were constructed as described [27]. The coding regions of ITGB4 and Y1494A mutant were subcloned into the pEGFP-C2 expression vector to produce the pEGFP-C2-ITGB4 and Y1494A mutant constructs. PC3 or HEK293 cells at 70% to 80% confluence were transfected with the expression vectors for 24 h by use of Lipofectamine 2000 (Invitrogen, 11668–019) according to the manufacturer's instructions. Then cells were harvested and analyzed by western blot or fluorescence assay.

### RNA interference

Specific siRNA against ANXA7 was designed and custom-synthesized by Invitrogen; ITGB4 siRNA (sc-35678) and scramble RNA (sc-37007) were obtained from Santa Cruz Biotechnology. PC3 cells at 70% to 80% confluence were transfected with 40 nM siRNA against ANXA7, ITGB4 and scramble siRNA with Lipofectamine 2000 (Invitrogen, 11668–019) according to the manufacturer's instructions. Then cells were harvested and analyzed by western blot.

### Capillary tube formation assay

The capillary-like tube formation assay was performed as previously described [66]. Matrigel was added to 24-well plates and allowed to polymerize for 1 h at 37°C. Human umbilical vein endothelial cells (HUVECs) were seeded on 24-well plates at 4–5 × 10^4^ cells/well in M199 medium, treated with or without SEC in the presence FGF-2 and serum, then incubated at 37°C for 24 h. Tube formation was observed under an inverted-phase contrast microscope (Nikon, Tokyo). The degree of tube formation was quantified by measuring the number of tubes in random fields from each well using the ImageJ.

### *In vivo* tumor assay of chick embryo chorioallantoic membrane (CAM)

Fertilized chicken eggs were incubated at 37.8°C with 60% relative humidity. On embryonic day 10, a silicone ring with a 5-mm inner diameter was placed on the CAM, and 6 million A549 cells in 20 μl of medium were seeded into this silicone ring. Four separate groups of 5 eggs each were divided. On day 2, SEC was deposited at 0.2 μmol (low dose) or 0.5 μmol (high dose) per egg every 2 days. 5FU (0.5 μmol) treatment was a positive control. At 6 days after SEC and 5FU treatment, the CAM and tumor were sampled. Tumor size-matching was based on tumor volume calculation: length × width × width × 0.5. Tumor tissues were fixed in 4% paraformaldehyde, snap-frozen in optimal cutting temperature embedding medium (Tissue-Tek, 4583), and cut into 9-μm cryosections.

### Angiogenesis assay of CAM *in vivo*

Fertilized chicken eggs were incubated at 37.8°C with 60% relative humidity. On embryonic day 10, SEC (0.2 μmol/20 μl and 0.5 μmol/20 μl) soaked in the gelatin sponge was applied to the CAM. CAMs were treated with SEC and DMSO as the vehicle control. Eggs were incubated for a further 6 days. At the end of the incubation, the CAM zones around the gelatin sponge were photographed and analyzed by using the Image-Pro Plus.

### Statistical analysis

Data are presented as mean ± SEM and were analyzed by one-way ANOVA with SPSS v11.5 (SPSS Inc., Chicago, IL). *P* < 0.05 was considered statistically significant. Mean values were derived from at least 3 independent experiments.

## SUPPLEMENTARY MATERIALS FIGURES AND TABLES


