# Supplementary material for: A small molecule induces integrin β4 nuclear translocation and apoptosis selectively in cancer cells with high expression of integrin β4
Source: Oncotarget. 2016 Feb 23;7(13):16282–96. doi: 10.18632/oncotarget.7646 (PMC4941314; doi:10.18632/oncotarget.7646)
Supplement: Supplementary file 1 [file oncotarget-07-16282-s001.pdf]

A small molecule induces integrin β4 nuclear translocation and apoptosis selectively in cancer cells with high expression of integrin β4

Supplementary Materials

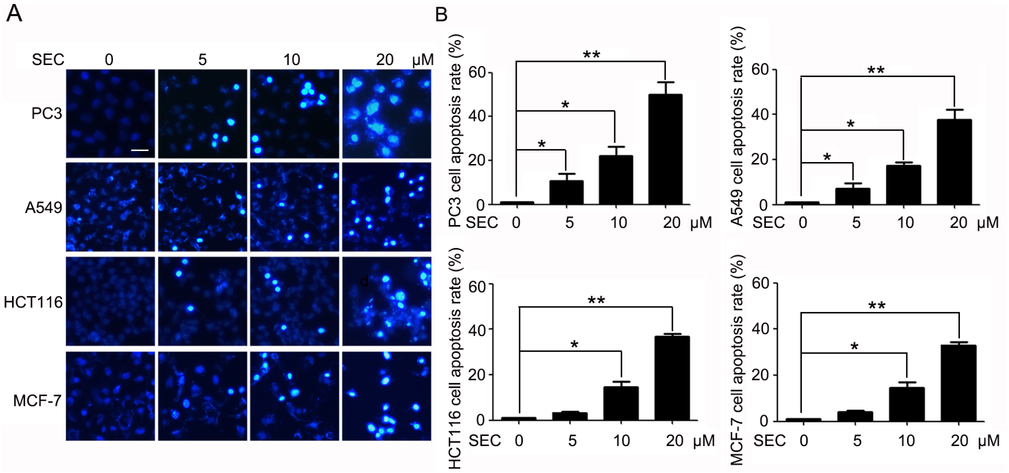

Supplementary Figure S1: Apoptosis of PC3, A549, HCT116, MCF-7 cells treated with SEC. (**A**) Fluorescent images of PC3, A549, HCT116, MCF-7 cells stained by Hoechst 33258 with SEC at the indicated concentrations for 24 h. (**B**) Quantitative statistics for apoptosis in PC3, A549, HCT116 and MCF-7 cells. Bar, 16 μM. Data are mean ± SEM; $n = 3$; $*p < 0.05$; $**p < 0.01$.

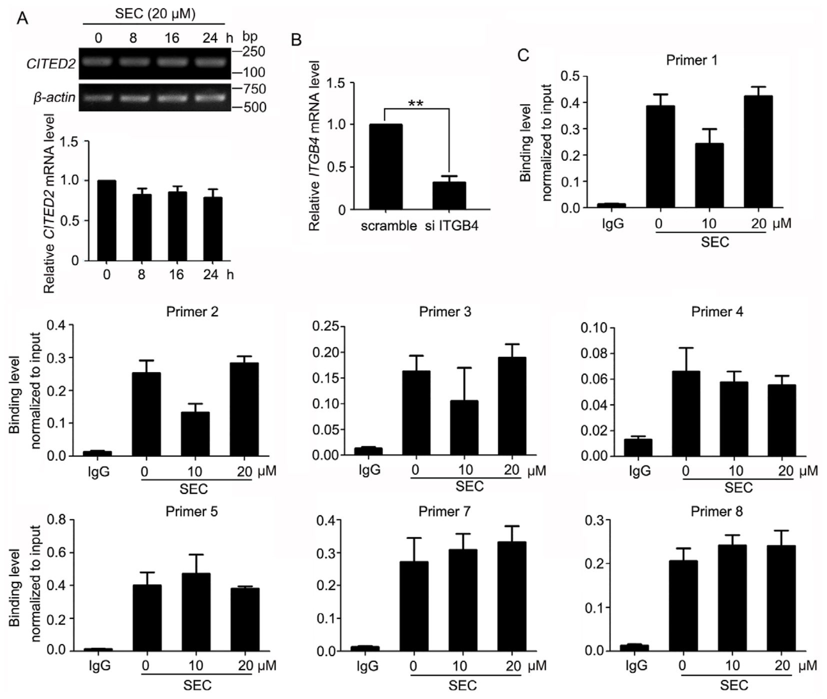

Supplementary Figure S2: Confirmation of a gene microarray result and ChIP-qPCR analysis. (**A**) RT-PCR analysis of mRNA levels of *CITED2* treated with SEC (20 μM) for indicated times. (**B**) qPCR analysis confirmed efficient knockdown of ITGB4 with *ITGB4* siRNA treatment for 24 h. (**C**) ChIP-qPCR analysis the effects of SEC treatment on the binding of ITGB4 to *ATF3* promoter using other 7 pair of primers, and the stastical analysis shows no significance. Data are mean ± SEM; $n = 3$; $**p < 0.01$.

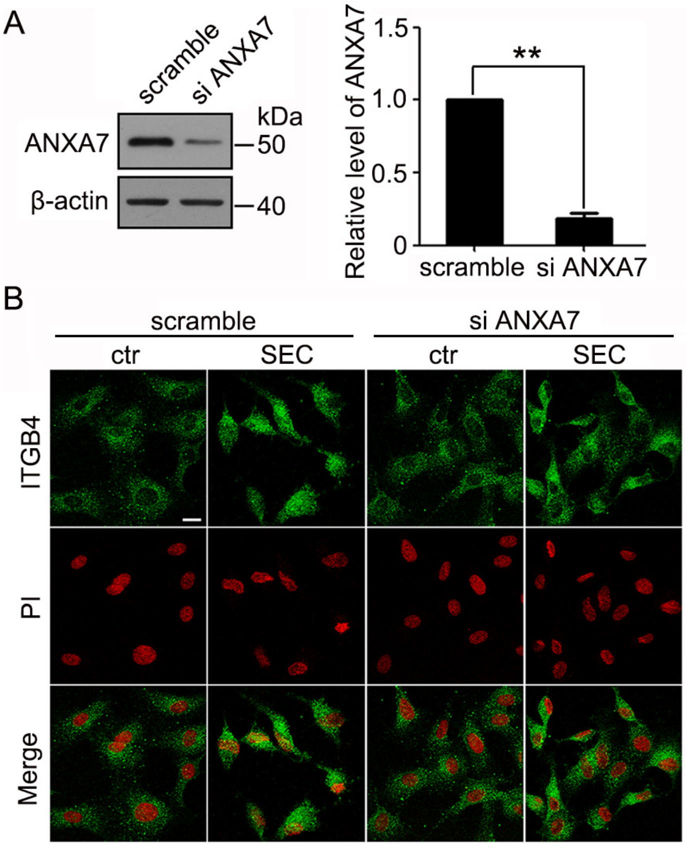

Supplementary Figure S3: Inhibitory efficient of ANXA7 by siRNA and broader visual field for observation of ITGB4 nuclear translocation. (**A**) Western blot analysis confirmed efficient knockdown of ANXA7 with ANXA7 siRNA treatment for 24 h. (**B**) Immunofluorescence images in broader visual field showed inhibition of ANXA7 by siRNA reduced ITGB4 nuclear accumulation induced by SEC. Nuclei were labeled with propidium iodide (PI). Images are representative of 3 independent experiments. Bar, 16 μM. Data are mean ± SEM; $n = 3$; $^{**}p < 0.01$.

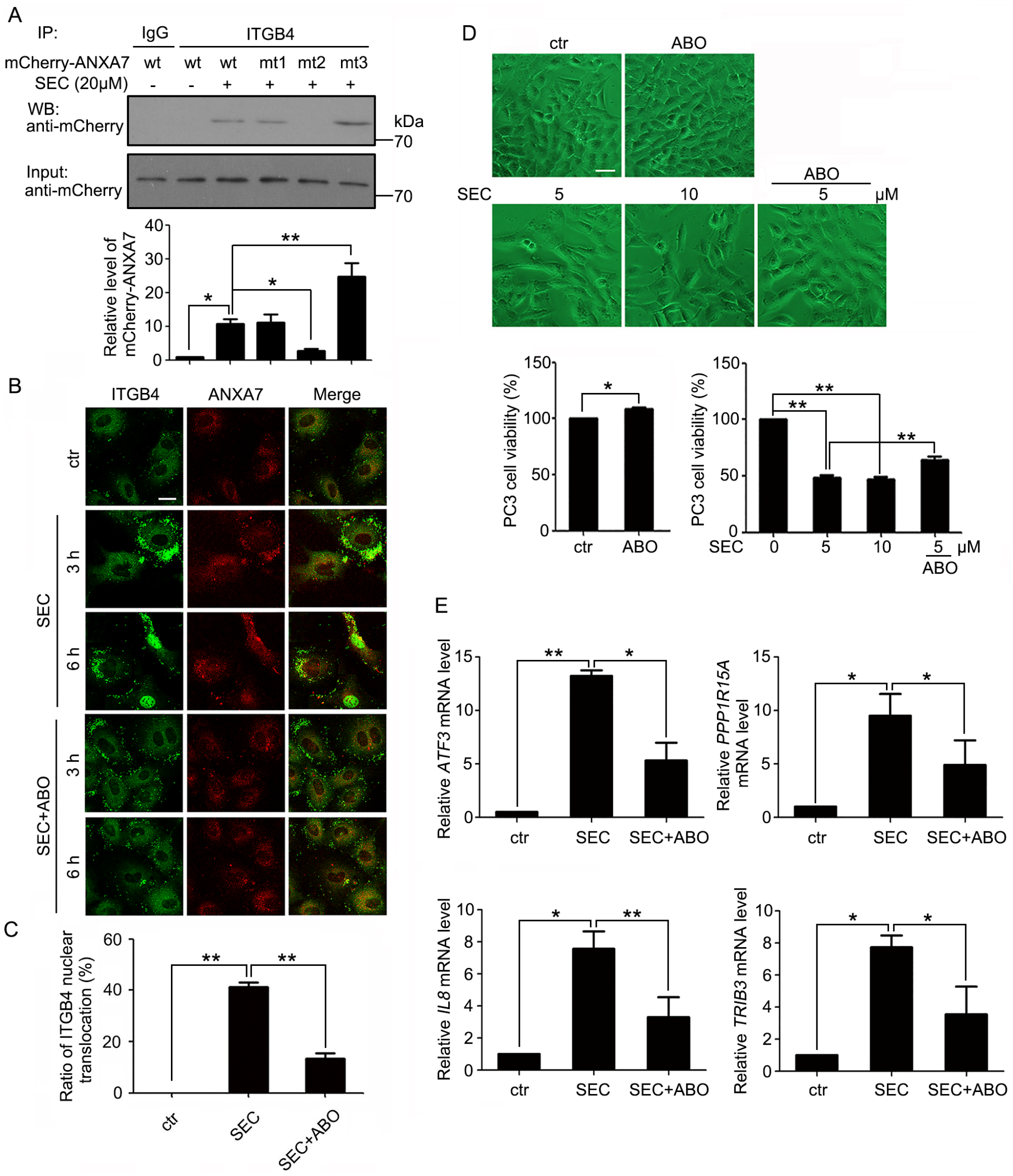

Supplementary Figure S4: Effect of ANXA7 GTPase activity on ITGB4 nuclear translocation and cell viability. (**A**) PC3 cells were transfected with mCherry-ANXA7-wt (wild type), -mt1 (T275A), -mt2 (T286A) and -mt3 (T286D) (mutants) for 48 h, then treated with 20 μM SEC for 24 h with 1% FBS. ITGB4 antibody was used in co-immunoprecipitation (co-IP). Western blot (WB) analysis of immunopreciates with mCherry antibody and quantification of co-immunoprecipitated mCherry-ANXA7-wt, -mt1 (T275A), -mt2 (T286A) or -mt3 (T286D). (**B**) Immunofluorescence assay of the co-localization of ANXA7 and ITGB4 treated with SEC (20 μM) with or without ABO (50 μM) for 3 and 6 h. ITGB4 was co-localized with ANXA7 at both 3 h and 6 h, and ITGB4 appeared in the nucleus at 6 h. Bar, 16 μM. (**C**) The quantification of ITGB4 nuclear translocation for Figure 4D in A549 cells incubated with SEC (20 μM) with or without ABO (50 μM) for 12 h. (**D**) Effects of SEC and ABO (50 μM) on PC3 cell morphology and viability at the indicated concentrations of SEC for 24 h. Bar, 32 μM. (**E**) qPCR analysis of *ATF3, PPP1R15A, IL8* and *TRIB3* mRNA levels with SEC treatment with or without ABO for 24 h. Images are representative of 3 independent experiments. Data are mean ± SEM; $n = 3$; $*p < 0.05$; $**p < 0.01$.

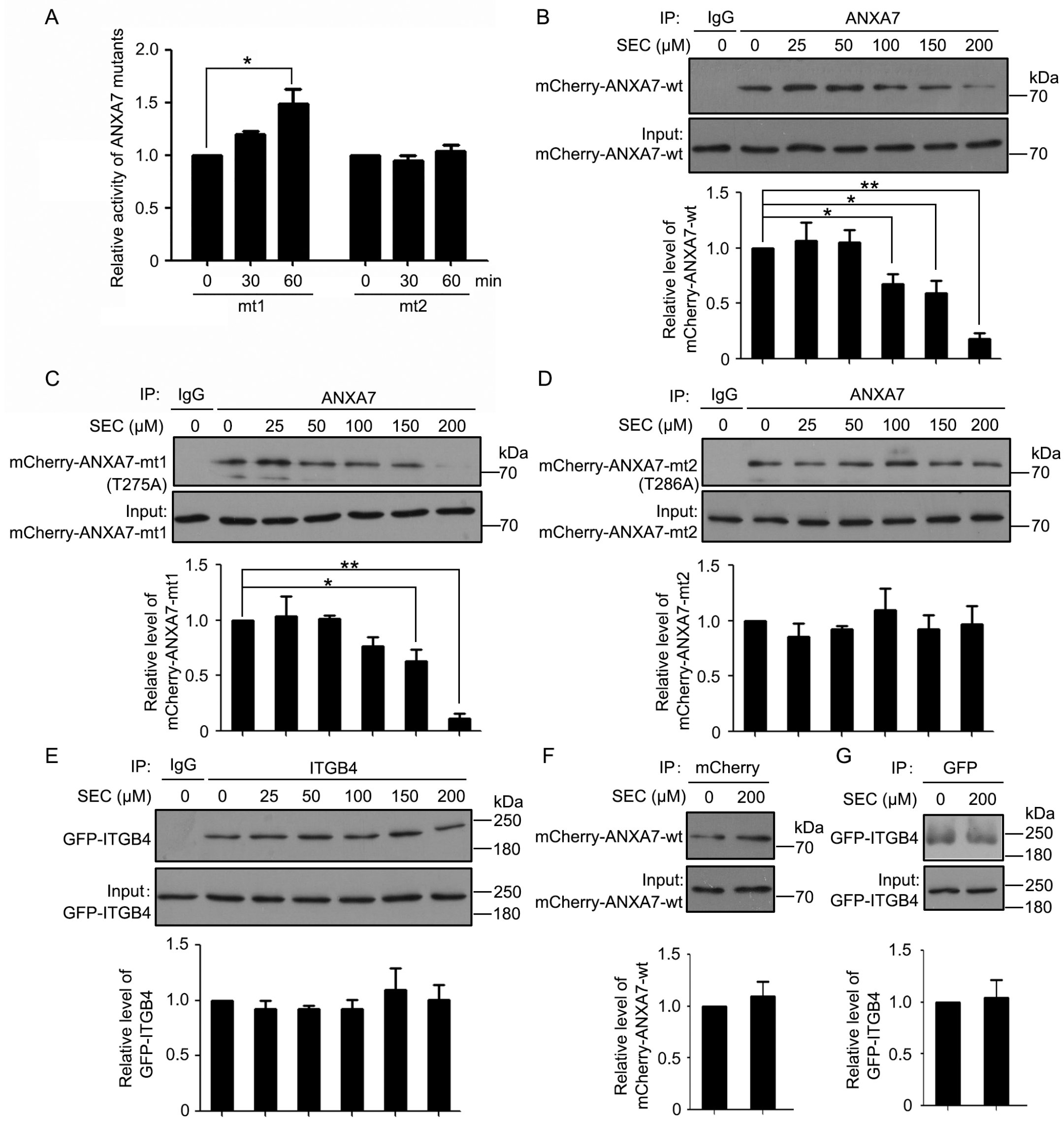

Supplementary Figure S5: SEC increases ANXA7-mt1 (T275A) GTPase activity and directly binds to ANXA7 rather than ITGB4. (**A**) Effect of SEC (20 μM) on ANXA7-mt1 (T275A) and -mt2 (T286A) GTPase activity at indicated times. HEK293 cells were transfected with plasmids coding for mCherry-ANXA7-wt, -mt1 (T275A) and -mt2 (T286A), and GFP-ITGB4 for 48 h. WB analysis of IP with ANXA7 antibody showed the dose effect of SEC on the binding of (**B**) mCherry-ANXA7-wt, (**C**) -mt1 (T275A) and (**D**) -mt2 (T286A) to its specific antibody. (**E**) WB analysis of IP with ITGB4 antibody showed the dose effect of SEC on the binding of ITGB4 to its specific antibody. WB analysis of IP (**F**) with mCherry antibody showing unchanged level of immunoprecipitated mCherry-ANXA7-wt with SEC treatment and (**G**) with GFP antibody showing unchanged level of immunoprecipitated GFP-ITGB4 with SEC treatment. Data are mean ± SEM; $n = 3$; *$p < 0.05$; **$p < 0.01$.

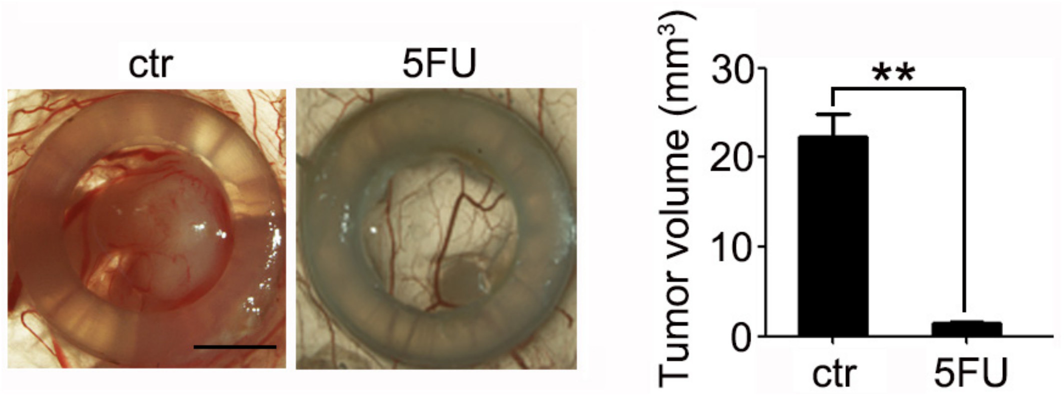

Supplementary Figure S6: Biomicroscopy imaging of control and 5FU (0.5 μM/egg) treated tumors. Tumor volumes were quantified. Bar, 1.5 mm. $n = 5$. Data are mean ± SEM; $^{**}p < 0.01$.

Supplementary Table 1: The primers for PCR used in microarray confirmation

Primers	Sense	Anti-sense
CITED2	5′-CAGAGTGAGCTGTTGACTCG-3′	5′-ACATAAGGGAGGTGGGTGAA-3′
ATF3	5′-TTGCAGAGCTAAGCAGTCGTGGTA-3′	5′-ATGGTTCTCTGCTGCTGGGATTCT-3′
PPP1R15A	5′-TGAGACTTCTGCTTCCACAC-3′	5′-CCTCACTATCCACATCCTCA-3′
IL8	5′-TCTCTTGGCAGCCTTCCTGATTTC-3′	5′-GTGTGGTCCACTCTCAATCACTCT-3′
TRIB3	5′-CCAAACCTTCAGTGCCTTCC-3′	5′-GTTGTCAGCTCAAGGATGCC-3′

Supplementary Table 2: The primers for PCR used in ChIP

Primers	Sense	Anti-sense
Primer 1	5′-GGGTGGTCTGAGTGAGGTC-3′	5′-TGGGGTAGAAACTAGAGGTGT-3′
Primer 2	5′-TGTTTTGTGGAGGACGGTCT-3′	5′-AGGAGGGAGAACACGTGAAG-3′
Primer 3	5′-CTTCACGTGTTCTCCCTCCT-3′	5′-AGGTGGAGGAGTGTTTGCAT-3′
Primer 4	5′-TCGCTTTACTTTTCGCCCTG-3′	5′-ATGCAAACACTCCTCCACCT-3′
Primer 5	5′-GTGCCTACTGTGTGATGCTG-3′	5′-ACTTCTTCGCTTTGGAACGG-3′
Primer 6	5′-CAAACCCAAATCCCCTGCTC-3′	5′-AGCGATCTCCAGGAATGACA-3′
Primer 7	5′-TGGGAGGCCTAGTAATCATGG-3′	5′-ATCATTGTACCTCCCCGTGG-3′
Primer 8	5′-ACTTGCTCACACTCCCAGAA-3′	5′-AGCAAATTTTCTCGGGGCAG-3′
